# Hypoxia-Mediated Decrease of Ovarian Cancer Cells Reaction to Treatment: Significance for Chemo- and Immunotherapies

**DOI:** 10.3390/ijms21249492

**Published:** 2020-12-14

**Authors:** Aleksandra Klemba, Lubomir Bodnar, Halina Was, Klaudia K. Brodaczewska, Gabriel Wcislo, Cezary A. Szczylik, Claudine Kieda

**Affiliations:** 1 Laboratory of Molecular Oncology and Innovative Therapies, Military Institute of Medicine, 04-141 Warsaw, Poland; hwas@wim.mil.pl (H.W.); kbrodaczewska@wim.mil.pl (K.K.B.); 2 College of Inter-Faculty Individual Studies in Mathematics and Natural Sciences, University of Warsaw, 00-927 Warszawa, Poland; 3 Department of Oncology and Immunooncology, Hospital Ministry of the Interior and Administration & Warmia and Masuria Oncology Centre, 10-228 Olsztyn, Poland; lubo@esculap.pl; 4 Department of Oncology, University of Warmia and Masuria, 10-228 Olsztyn, Poland; 5 Department of Oncology, European Health Centre, 05-400 Otwock, Poland; gabrielwcislo@yahoo.pl (G.W.); cszczylik@wp.pl (C.A.S.); 6 Centre of Postgraduate Medical Education, 01-809 Warsaw, Poland

**Keywords:** angiogenesis, chemotherapy, hypoxia, immunosuppression, immunotherapies, microenvironment, ovarian cancer

## Abstract

Hypoxia, a common factor ruling the microenvironment composition, leads to tumor progression. In this hypoxic context, cytokines and cells cooperate to favor cancer development and metastasis. Tumor hypoxia is heterogeneously distributed. Oxygen gradients depend on the vicinity, functionality of blood vessels, and oxygen ability to diffuse into surrounding tissues. Thus, the vasculature state modulates the microenvironment of the tumor cells. Cells sense and react to small variations in oxygen tension, which explains the lack of tumor cells’ unicity in their reaction to drugs. Ovarian cancers are highly hypoxia-dependent, ascites worsening the access to oxygen, in their reactions to both chemotherapy and new immunotherapy. Consequently, hypoxia affects the results of immunotherapy, and is thus, crucial for the design of treatments. Controlling key immunosuppressive factors and receptors, as well as immune checkpoint molecule expression on tumor, immune and stromal cells, hypoxia induces immunosuppression. Consequently, new approaches to alleviate hypoxia in the tumor microenvironment bring promises for ovarian cancer immunotherapeutic strategies. This review focuses on the effects of hypoxia in the microenvironment and its consequences on tumor treatments. This opens the way to innovative combined treatments to the advantage of immunotherapy outcome in ovarian cancers.

## 1. Ovarian Cancer to Date 

Ovarian cancer (OC) is the most aggressive gynecological malignancy. It accounts for ci. 240,000 female cancer cases and is responsible for ci. 150,000 cancer-related deaths. More than three-fourths of patients suffer from disease recurrence within 24 months after initial treatment [1].

One of the main problems for OC treatment is its late diagnosis—ci. three-fourths of cancer cases are diagnosed at stage III/IV with disseminated disease. The early discovery of OC is rendered difficult due to the lack of specific symptoms and of effective diagnostic tools. Unfortunately, measurement of the serum CA-125 marker concentration, transvaginal ultrasound examination, or both have not provided clinically relevant evidence to detect OC in early stages, and the median age of diagnosis of OC is 63 years [2]. Abdominal pain and distension for 3–4 months are frequently attributed in women to irritable bowel syndrome and not a tumor mass present in the pelvis [3,4].

Based on physical examination and imaging, OC can be suspected, and an exploratory laparotomy is typically performed to obtain histopathological confirmation of the diagnosis, staging, and tumor debulking. To date, treatments are mainly based on aggressive cytoreductive surgery in combination with platinum-based and taxane-based chemotherapy. Other drugs (pegylated liposomal doxorubicin, topotecan, and gemcitabine) are used as the second-, third- and subsequent lines of treatment of recurrent OC [5]. Cytoreductive surgery usually offers successful removal at the level of more than 75% of the initial tumor mass, with residual tumor not larger than 2 cm further reduced to 1 cm. Resection methods, as well as the mode and duration of chemotherapy administration, have considerably evolved since the 1990s [4,6,7].

Lack of residual disease (R_0_) after primary debulking surgery (PDS) is considered the most important prognostic factor [8]. In advanced OC, two randomized phase III studies compared the effectiveness of first-line neoadjuvant chemotherapy (NACT) followed by surgery, to the standard therapeutic approach (i.e., PDS) followed by adjuvant chemotherapy. Both methods showed similar overall survival (OS) and progression-free survival (PFS). Significantly lower operational mortality was observed in the NACT first-line group [9,10]. However, both studies were criticized for low survival and R_0_ rates, and the choice between both modalities remains controversial [11,12].

The standard first-line chemotherapy regimen for OC is intravenous carboplatin (area under the curve 5–6) and paclitaxel (175 mg/m^2^) every three weeks [13]. Modifications of both carboplatin and paclitaxel administration advise a weekly dose-dense as an alternative [14,15,16].

Search for new drugs has resulted in a significant prolongation of the median PFS. This was mainly obtained when immunotherapy was introduced as anti-angiogenic monoclonal antibodies. Bevacizumab, an anti-vascular endothelial growth factor (VEGF) antibody, has been largely tested for its potential as an adjuvant strategy. It was finally observed that, despite the wide action of the monoclonal antibodies, the immunotherapy approach did not produce a noticeable improvement in OS. Indeed, bevacizumab did not improve OS when combined with standard paclitaxel/carboplatin chemotherapy [17,18]. The benefit of adding bevacizumab was observed to the greatest extent in FIGO III patients with residual disease larger than 1 cm after PDS or in FIGO IV patients [18]. These findings led to the reimbursement of bevacizumab with standard paclitaxel and carboplatin chemotherapy in high-risk patients in many countries.

Another issue is the administration of cisplatin and paclitaxel by intraperitoneal injection. Some benefit of this form of therapy was observed with a significant prolongation of median OS in patients with residual disease below 1 cm after PDS [19]. Despite controversial results, chemotherapy regimen has become the standard. Recently, after the GOG252 study questioning the effectiveness of intraperitoneal vs. intravenous chemotherapy combined with immunotherapy (bevacizumab), the enthusiasm for this treatment formula has significantly diminished [20].

Hyperthermic intraperitoneal chemotherapy (HIPEC) is one of the OC treatment optimizations. A Dutch randomized study using HIPEC during interval debulking surgery (IDS) after NACT showed significant prolongation of PFS and OS in the HIPEC-treated arm as compared to the IDS control [21]. However, those results were not confirmed in the Korean trial [22].

Recurrence of OC is incurable in about three-fourths of women with advanced-stage disease. For relapses susceptible to standard platinum, re-challenge with platinum doubled chemotherapy is recommended. Then, maintenance strategies have been developed to delay disease progression. Phase III studies maintaining bevacizumab have shown significant benefit in the disease control indexes in platinum-sensitive and platinum-refractory OC [23,24].

Non-platinum monotherapy is usually used to treat patients with platinum-resistant recurrent OC. This includes pegylated liposomes to deliver and release doxorubicin and weekly administration of topotecan, paclitaxel, gemcitabine, or docetaxel. However, this type of treatment is not very effective, as the overall response rate ranges from 10% to 35% with a relatively short response (less than eight months) [25]. 

Treatment with PARP inhibitors has been implemented as maintenance treatment, as well as in recurrent OC, exploiting the inherent disorders DNA repair in approximately 50% of OCs via BRCA1, BRCA2 mutations, or homologous recombination associated genes, as well as inactivation via methylation [6,26].

The mechanisms of chemoresistance are only partially elucidated. One of the most important factors affecting the resistance of cancer cells in the tumor microenvironment (TME), with its key modulator-oxygen level. This parameter was neither investigated nor taken into account when the drugs were approved for clinical applications.

Considering the new strategies that have been approached and that are leading the search for combined therapies, it appears that hypoxia alleviation is one of the main challenges.

As OCs typically display an acute hypoxic state, the need for hypoxia compensation appears in all treatment attempts, indicating that the combined treatments (although more promising) might be senseless if the TME is not considered as the first-line strategy, not allowing the second-line combinatorial attempts to gain any efficacy.

## 2. Hypoxia as a Key Microenvironment Modulator in Ovarian Cancer

Hypoxia, oxygen partial pressure lower than its physiological value, appears within the growing tumor and is one of the most important factors shaping the TME. Several studies show that it influences cellular processes, as angiogenesis and epithelial-to-mesenchymal transition (EMT), and cell characteristics (such as the acquisition of stem-like features) with deep consequences on the activity and effectiveness of anti-cancer drugs [27]. In cancer, as in other hypoxia-dependent diseases, low oxygen tension favors tumor growth and the development of immunosuppression. Hypoxia switches the tumor from benign to aggressive, and concomitantly, turns on the angiogenesis and its pathologic characteristics. The latter is a major factor in tumor development. Tumor angiogenesis is a condition that favors the growth and dissemination of the tumor cells for its lack of effectiveness in reestablishing the physiologic pO_2_, which is also responsible for immunosuppression and tolerization of anti-tumor cytotoxic immunocompetent cells [28]. 

Low oxygen partial pressure activates hypoxia-dependent signaling pathways mainly via stabilization of hypoxia-inducible factor-1α (HIF-1α), the presence and mechanism of which was first described by Gregg L. Semenza [29]. Under normoxia, two prolyl residues of HIF-1α are hydroxylated by prolyl hydroxylase 2 (PHD2), which was found by P. Kaelin and W. G. Ratcliffe to act as an oxygen sensor [30]. Hydroxylated HIF-1α can bind to Von Hippel Lindau protein (pVHL), as P. Radcliffe described [31], and such a complex is ubiquinylated before proteasomal degradation [32]. In hypoxic conditions, enzymes modifying HIF-1α are inactive, and unmodified HIF-1α translocates to the nucleus, where it binds to HIF-1β forming HIF-1 heterodimer. HIF-1complex functions as a transcription factor and locates to its downstream targets in the genome: The hypoxia-response elements (HREs) [29,31,33]. The other HIF mediating the hypoxic response is HIF-2 [34], which shares structural similarity with HIF-1α, but it regulates different downstream targets [35].

A consequence of intratumor hypoxia is a switch leading to the development of chaotic pathological vessels, which are inefficient in compensating hypoxia. This maintains the pro-angiogenic hypoxic state, which influences the physiology of the tumor cells and overall stroma. Typically, cancer cells in a hypoxic microenvironment may acquire a mesenchymal phenotype (*via* EMT), leading to increased cell mobility and the ability to metastasize [36]. It also significantly affects cell metabolism contributing to their chemoresistance [37]. Hypoxia causes acidosis via the production of high amounts of lactic acid in the cancer microenvironment [38]. 

Active migration of cancer cells into (intravasation) and out of (extravasation) the blood or the lymphatic vessels is also regulated by HIF-1 [39]. Its action in cancer cells affects cancer-endothelial and endothelial cell interactions [40,41,42]. Hypoxia-induced angiogenesis is essential for those processes as it produces pathological, “leaky” vessels. Vascular permeability is controlled by HIF-1-dependent genes, such as vascular endothelial growth factors (VEGFs), metalloproteinase 2 (MMP2), angiopoietin 2, and urokinase receptor (UPAR), that act on the disruption of vascular wall integrity and facilitate cancer cell migration [43]. HIF-1 signaling strongly modulates endothelium properties by modifying the characteristics of cell adhesion, coagulation, and endothelial permeability, as well as growth [43].

### Prognostic and Predictive Value of HIF-1α

The exact prognostic and predictive values of HIF-1α expression remain to be fully understood, as clinical data are often inconsistent. High HIF-1α expressing tumors presented higher response rates to postoperative paclitaxel/carboplatin combined chemotherapy. Patients with such tumors after suboptimal resection (of stage III/IV tumors) and indicated for postoperative combined chemotherapy, showed significantly better survival [44]. HIF-1α was also strongly expressed in tumors of patients with longer PFS [45]. On the other hand, strong HIF-1α expression was a significant indicator of shorter OS and shorter median progression-free interval (PFI). Moreover, the overall PFI of patients with (1) tumors displaying strong HIF-1α expression and (2) suboptimal cytoreduction at primary surgery, was significantly worse [46]. A meta-analysis of 31 OC showed that the level of HIF-1α expression correlated with worse OS, worse disease-free survival (DFS), progression-free survival (PFS), cancer-specific survival (CSS), relapse-free survival (RFS), and worse metastasis-free survival (MFS). Such associations were found neither for HIF-2 expression nor other investigated parameters [47].

## 3. Chemotherapeutic Treatment of Ovarian Cancer in Hypoxia

### 3.1. Platinum-Based Chemotherapeutics

Low expression of HIF-1α protein in OC patients correlated with response to cisplatin treatment [48]. Treatment of some OCs with cisplatin resulted in elevated HIF-1α expression in cells in vitro [49]. Interestingly, cisplatin decreased the level of HIF-1α in cisplatin-sensitive OC but not in cisplatin-resistant ones. Both types of cells (resistant and sensitive ones) showed enhanced cisplatin sensitivity after HIF-1α knockdown or pharmacological promotion of HIF-1α degradation [50]. Cisplatin strongly reduced the protein levels of the HIF-1 co-activators p300 and CREB-binding protein (CBP) under hypoxia [51]. Hypoxia during treatment was the most important factor determining chemoresistance, as opposed to hypoxia exposure prior to treatment [52]. This effect occurred irrespectively of *TP53* status [53]. The exact mechanisms as to how hypoxia contributes to chemoresistance is still a matter of intensive research. A hypoxic microenvironment was shown to affect the content of exosomes in a way that hypoxic exosomes (Hex) contain more oncogenic proteins than normoxic ones (Nex). When cultured in the presence of Hex, cancer cells displayed both higher survival and higher metastatic potential after cisplatin treatment [54]. Other processes involved in resistance to cisplatin might be HIF-1α-mediated decrease cisplatin-induced autophagy [55]. Knockdown of HIF-1α in SKOV3 and A2780 OC cells promoted autophagy and decreased the PI3K/AKT/mTOR signaling pathway [56]. HIF-1α with the histone deacetylase inhibitor 4 (HDAC4) possibly mediate p53-RAS crosstalk that actively regulates resistance to cisplatin via apoptosis and autophagy [57]. Although not fully elucidated, the mechanism of *Emblica officinalis’* extract action, known to inhibit the growth of OC cells, was shown to inhibit HIF-1α and activate autophagy [58]. Moreover, the extract from the natural compound Chansu (bufalin) inhibited mTOR, and consequently, decreased HIF-1α, resulting in a lower rate of cancer cell growth and migration [48].

Taking into account the variability of the cells and pathways that are affected by the chemotherapeutic drugs and immunotherapeutic tools, as exemplified in Table 1, the need for a combined approach of treatments arises. It shows that the microenvironment might be strongly modified by immunomodulators designed to neutralize the immune checkpoints activity, lowering their barrier effect towards anti-cancer cells chemotherapeutics.

In this line, HIF-1α emerges as an important target to improve cancer cells’ sensitivity to cisplatin, for its activation effect on PI3K/AKT and MAPK/ERK pathways [59]. SB202190, a MAPK inhibitor, helped to sensitize OC cells to glucose analogs (2-deoxy-glucose (2-DG) and D-allose) and further to cisplatin treatment. This effect involved a decrease in HIF-1α accumulation [60]. Blocking the Rho/ROCK pathway also increased the effectiveness of cisplatin treatment via HIF-1α inhibition in OC cells [61].

When CoCl_2_-induced resistant OC cells were treated with noscapine (a small opioid molecule and inhibitor of HIF-1α) upon cisplatin treatment, the level of apoptosis and proliferation inhibition increased. Noscapine-mediated inhibition of HIF-1α activity occurs by increased proteasomal degradation [62]. Sulforaphane decreases HIF-1α level promoting an anti-angiogenic response and elevates anti-cancer activity (p53, redox effector factor (ARE), interferon regulatory factor 1 (IRF-1), Pax-6, and X-responsive elements (XRE)). Moreover, it targets carbonyl anhydrase 9 (CA IX), an enzyme which is an important HIF-1α-downstream effector as it catalyzes the reversible hydration of carbon dioxide to bicarbonate ions and protons [63,64]. It protects cancer cells from hypoxia-induced pH imbalance, facilitating their migration and invasion [65]. 

MicroRNAs as noncoding RNAs are important modulators that can specifically target the gene coding for HIF-1α. As such, a low level of miR-199a expression was observed in OC tumors (as compared to normal tissues) and was associated with shorter survival. It downregulates HIF-1α, consequently, its lack affects the resistance level, as shown for cisplatin in OC cells [66].

Several proteins were identified as positive regulators of HIF-1α. SENP-1 upregulated HIF-1α expression by deSUMOylation and decreased hypoxic reaction to cisplatin treatment [67]. HIF-1α was also activated by exposure to recombinant human FSH (rhFSH) [68]. The Rab GTPase (Ras-related protein Rab25) was a positive regulator of HIF-1α in several OC cell lines, conferring increased resistance to platinum derivatives. This upregulation was based on *de novo* HIF-1α synthesis via ERRB2/ERK1 and p70S6K/mTOR pathways [69].

Targeting the formyl peptide receptor (FPR) and toll-like receptor 9 (TLR9) sensitized OC cells to cisplatin [70]. Indeed, hypoxia upregulated both molecules, and their blockage significantly reduced the cisplatin-induced inhibition. 

Mitochondrial metabolism plays a key role in cancer cell adaptation to a hypoxic microenvironment. Hypoxia-induced reactive oxygen species (ROS) trigger mitochondrial fission, resulting in resistance to cisplatin by decreasing levels of p-Drp1 and Mnf1, which are key proteins regulating this process [53].

Resistance to carboplatin was shown to be cysteine-dependent. Some OC cells exhibited a strong dependency on their chemoresistance on cysteine levels in hypoxia [71,72].

Although mechanistically related to the hypoxic/physioxic balance, those treatments were hardly studied in instances that took into account the pO_2_ values in the microenvironment. Consequences on the degree of vascularization and on vessels functions are direct and contribute to modulate the composition of the microenvironment for its response to treatment. 

### 3.2. Alkylating Agents

Alkylating agents prevent the proper formation of a DNA double helix by adding an alkyl group to guanine residues. Cyclophosphamide, one of the first drugs applied in OC treatment, is now rarely applied in clinics. It potentiates the cytotoxicity of genetically modified tumor-infiltrating macrophages (transduced with CYP2B6 gene under hypoxia-responsive promotor). This gene encodes a prodrug-activating enzyme, human cytochrome P450 2B6 [73]. Melphalan is an alkylating agent rarely applied in OC treatment. It increases the oxidative stress response mediated by VEGF/IL8-signaling, but it was not investigated in hypoxia in the context of OC [74].

### 3.3. Mitosis Inhibitors

Mitosis inhibitors are mostly compounds of plant origin. They inhibit cancer cell division by binding to tubulin and inhibiting its polymerization into microtubules. An initial study on the influence of paclitaxel and docetaxel on OC cells in hypoxia showed that they affect neither HIF-1α nor VEGF expression [51]. However, other studies show a possible role of HIF-1α in cancer cell resistance to paclitaxel. Huang and co-workers showed that HIF-1α expression induced by hypoxia contributes to chemoresistance via G0/G1 arrest [75]. Long-term exposure to the cytokine Epo, a glycoprotein secreted upon hypoxia that stimulates erythrocytes production, increased the OC cells’ resistance to paclitaxel. In hypoxia only, the treatment of Epo-stimulated OC cells increased the proangiogenic properties of A2780 cells [76], which indicates the involvement of HIF-1 activation. C-Src played an important role in hypoxia-mediated resistance to paclitaxel by decreasing the numbers of cells in G2/M, its inhibition blocked HIF-1α and reversed the resistance [77]. 

The treatment of OC, both in vivo and in vitro, with paclitaxel and sMEK1 helped overcome resistance to paclitaxel by inhibiting downstream target genes of the S6K/4E-BP pathway (i.e., HIF-1α and VEGF) [78].

Directly linked to the hypoxic status, albumin-bound paclitaxel (nab-paclitaxel) applied with topotecan in a metronomic schedule was able to significantly inhibit tube formation by endothelial cells [79].

Consequently, the mitosis inhibitors used in the treatments of OCs are clearly dependent on the hypoxic environment for their final activity through the modulation of their molecular targets.

### 3.4. Antibiotics 

Antibiotics are compounds derived from microorganisms that affect DNA replication by several cytotoxic mechanisms. Doxorubicin interacting with DNA inhibits the hypoxia-mediated activation of HIF-1, although this drug has no significant effect on the expression levels of HIF-1α. The expression of p300 and CBP were only weakly reduced [51]. The resistance of cancer cells to doxorubicin could be induced by short exposure to rhFSH [68]. 

### 3.5. Miscellaneous Antineoplastic Agents

**Figure 1:**
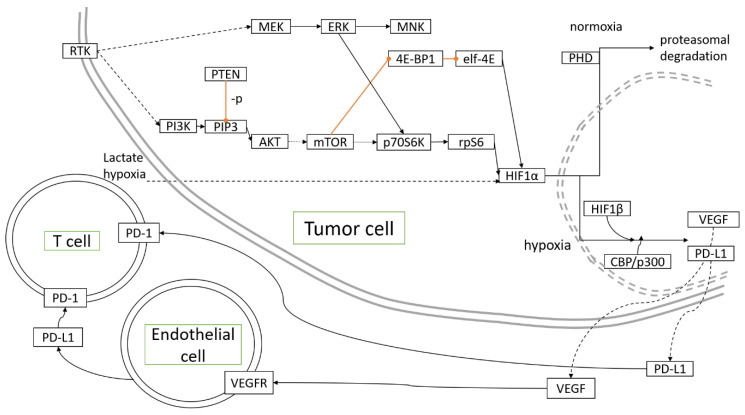
Molecular pathways activated and controlled by hypoxia in ovarian cancer development. 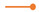 inhibition, 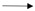 direct interaction/activation, 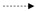 indirect interaction/activation.

Miscellaneous antineoplastic agents are drugs that decrease cancer cells growth through diverse mechanisms. Topotecan, a topoisomerase I inhibitor, inhibited HIF-1α, and increased hypoxic areas in tumors developing in mouse models of OC [80]. Metronomic application of topotecan appeared to reduce HIF-1α and VEGF expression [81], thus, acting as an angiogenesis-directed treatment as hypothesized [82]. Upon treatment with topotecan, topoisomerase I binding to HIF-1α mRNA was essential to restore p53 transcriptional activity, which participated in reversing hypoxia-mediated resistance to cisplatin and paclitaxel [83]. Figure 1 depicts the molecular pathways associated with hypoxia in OC development.

Table 2 points to the remarkable variability of the tumor treatment effects and their dependency upon the microenvironmental oxygen tension, which may explain a large part of the cancer hallmarks molecular mechanisms and their consequences.

## 4. Targeted Therapies of Ovarian Cancer in Hypoxia

### 4.1. Anti-Angiogenic Therapies

Anti-angiogenic therapies aim at interfering with the development of blood vessels in the tumor site [88]. Withdrawal of anti-angiogenic treatment resulted in tumor rebound accompanied by platelet infiltration. Focal adhesion kinase (FAK) in platelets played an important role in their migration into the tumor site. Combined therapy with anti-angiogenic agents and FAK inhibitors did prevent tumor rebound [89]. 

The strong effect of HIF-1 modulators cannot be dissociated from their effects on endothelial cells in the process of angiogenesis. Tumor angiogenesis-directed treatments in OC are particularly significant with regards to the hypoxic regulation of the entire microenvironment.

### 4.2. PARP Inhibitors

Poly (ADP-ribose)polymerases (PARPs) are a family of enzymes involved in the synthesis of poly(ADP-ribose) (PAR) chains from NAD+. Three of them (PARP-1, -2, and -3) have defined roles in DNA damage repair. PARP inhibitors (PARPi) can sensitize cells to a variety of DNA damaging agents. This is particularly true in the case of cells incompetent for homologous recombination repair (HRR) (e.g., BRCA1 and BRCA2 mutants). Therefore, a combination with cytotoxic chemotherapy or radiotherapy has been proposed as an approach for the treatment of HRR incompetent tumors. However, PARPis used in combination therapies often lead to normal tissue toxicity [90]. PARPi with cediranib (a small molecule inhibitor of VEGFR-2, platelet-derived growth factor receptor (PDGFR), and c-kit) showed better clinical effects than PARPis alone, regardless of BRCA1/2 status [91]. Cediranib confers sensitivity to PARPis by decreasing homology-directed DNA repair (HDR). This effect was hypoxia-dependent as cediranib induced hypoxia-related suppression HDR elements (BRCA1/2 and RAD51). But hypoxia-independent effects occurred through PDGFR inhibition, protein phosphatase 2A (PP2A), and E2F transcription factor 4 (E2F4)/RB transcriptional corepressor like 2 (RB2/p130) [92]. 

## 5. Hypoxia in Tumor Immune Microenvironment and Its Role in Immunotherapy of Ovarian Carcinoma

The majority of early immune therapies focused on potentiating T lymphocyte-mediated anti-tumor adaptive immunity. Treatments applying interleukin (IL)-2 or based on autologous T lymphocytes resulted in little or no clinical benefit. Much more promising results were obtained with so-called immune checkpoint inhibitors (ICIs), whose goal is to block T cell immune activity, namely, cytotoxic T lymphocyte-associated protein 4 (CTLA-4) and programmed cell death protein 1 (PD-1) [93].

In hypoxic cells, HIF-1α binds the HRE located in the proximal PD-L1 promoter. The functional consequences of the overexpression of PD-L1 on the surface of myeloid-derived suppressor cells (MDSCs) include the increased production of IL-6 and IL-10 under hypoxic conditions and significantly decreased the proliferation of CD8+ T cells. Taken together, these phenotypic changes are indicative of enhanced immunosuppression when MDSCs are exposed to hypoxia. Treatment with an anti-PD-L1 antibody significantly decreased both the expression of IL-6 and IL-10 and CD8+ T cells [94].

The activity of hypoxia-induced factors affects cancer immunosuppression in patients with OC. In a study of 21 OC patients, the mRNA expression of selected hypoxia-related genes and tumor-infiltrating leucocytes was assessed by flow cytometry to identify regulatory T cells, MDSC, and type 2 macrophages. The number of tumor-infiltrating leucocytes varied from 2% to over 50% of the total cell population. The heterogeneous immunological microenvironment in OC patients was confirmed by the relative proportions of suppressors cells. This complicates immune checkpoint therapies and may contribute to the low response rate. Clustering at the mRNA level revealed a small group with high expression of HIF target genes and increased expression of HIF-1α and HIF-2α proteins, which may increase their susceptibility to immunotherapy [95].

The establishment of modern immunotherapy in OC comes from a study by Disis et al. [96]. In this phase 1B study, avelumab (anti-PD-L1 antibody) used as a single-agent in 125 patients with previously relapsed platinum-resistant OC showed encouraging anti-tumor activity, with an objective response rate (ORR) of 9.6% and an acceptable toxicity profile. 

These results provided the basis for initiating the phase III JAVELIN Ovarian 200 study. It compared the activity of standard chemotherapy with pegylated liposomal doxorubicin (PLD) in patients with platinum-resistant OC vs. the combination of avelumab together with PLD or avelumab alone. Unfortunately, avelumab did not significantly increase the PFS or OS compared to standard PLD. The ORRs did not differ significantly between the arms and individual subgroups and were: 3.7%, 13.3%, and 4.2% for avelumab, avelumab plus PLD, and PLD monotherapy, respectively. PD-L1 status did not affect the median PFS [97].

The next randomized phase III JAVELIN Ovarian 100 study in previously untreated patients with locally advanced or metastatic (FIGO III/IV stage) OC compared the efficacy of avelumab with standard chemotherapy. Patients were randomized to three arms receiving: Carboplatin/paclitaxel (A); carboplatin/paclitaxel with the maintenance of avelumab in (B); or avelumab plus carboplatin/paclitaxel, followed by avelumab maintenance therapy (C). The hazard ratio, (HR with 95% CI) for PFS in the avelumab-treated patients was 1.43 (95% CI 1.051–1.946) and 1.14 (95% CI 0.832–1.565) for A and B, respectively. The median PFS was 16.8 months (95% CI 13.5-NE) and 18.1 months (95% CI 14.8-NE) in group B and C, respectively. In the control group, the medians (NE (18.2-NE)) were not reached. Subgroup analysis based on baseline characteristics and biomarkers (PD-L1, CD8, and BRCA mutations) failed to select subgroups that showed significant benefits. OS data were not published yet. The ORRs were: 30.4%, 36.0% and 30.4% for B, C and control, respectively. Avelumab treatment did not result in PFS prolongation (i.e., the primary endpoint was not reached compared to the control receiving standard chemotherapy) [98].

Those results had a direct impact on the decision of Merck and Pfizer, in March 2019, to discontinue another ongoing project [99], a randomized phase III trial of JAVELIN Ovarian PARP 100 [100] which planned to compare the efficacy and safety of avelumab in combination with standard paclitaxel/carboplatin chemotherapy or talazoparib, a PARPi.

Phase II study compared the efficacy of avelumab in combination with a class I selective HDAC—entinostat, to avelumab monotherapy in patients with advanced OC. Heavily pretreated patients (from 3–6 lines therapy) were randomly assigned in a 2:1 ratio to two arms—A: Avelumab (10 mg/kg, IV every 2 weeks) plus entinostat (5 mg, PO daily), or B: Avelumab plus placebo. Median PFS did not differ significantly between the groups, being 1.64 and 1.51 months (HR 0.90, 95% CI: 0.58–1.39; *p* = 0.31) for A and B, respectively. There were no significant differences in secondary endpoints (i.e., ORR (6% vs. 5%) and OS (median not reached vs. 11.3 months)) [101].

The activity of pembrolizumab, ICI, and anti-PD-1 antibody, was assessed in a phase II study, KEYNOTE-100, in patients with recurrent OC. Cohort A consisted of 285 patients who had previously received 1–3 treatment lines with a platinum-free interval or treatment-free interval (TFI) between 3 and 12 months. Cohort B included 91 patients who had previously received 4–6 previous lines with PFI/TFI ≥ 3 months. The ORRs were: 7.4% and 9.9% in cohorts A and B, respectively. The median response time was 8.2 months for cohort A and was not achieved for cohort B. The disease control rates (DCRs) were similar in both cohorts (37.2% and 37.4%). The median OS was not provided in cohort A, and in cohort B, was 17.6 months [102].

Preclinical studies data indicated a synergistic effect of ICIs with PARPis [103]. Patients with recurrent OC were enrolled in a single-armed phase II study evaluating the efficacy of ICI with PARPis. A total of 35 heavily pretreated patients, with a median of four prior lines of therapy, received olaparib 300 mg twice daily and durvalumab 1500 mg intravenously every four weeks. The ORR was 14% (5/35 patients). The DCR (PR + SD) was achieved in 71% (25/35 patients). The treatment increased the expression of IFNγ and CXCL9/CXCL10 in the tumor, as well as increased serum IFNγ/TNFα and expression of tumor-infiltrating lymphocytes, which may indicate the effectiveness of durvalumab/olaparib combination therapy in inducing an immune response in the tumor. Increased IFNγ expression was associated with improved PFS (HR 0.37 (95% CI 0.16–0.87); *p* = 0.023), while increased VEGFR3 levels were associated with worse PFS (HR 3.22 (95% CI 1.23–8.40); *p* = 0.017) [104].

The published results on the roles of avelumab, pembrolizumab and durvalumab are the first data from phase II and III studies to evaluate the role of ICIs in OC. No significant results of treatment improvement were achieved for ICIs, both in relapse and in the first-line treatment of OC. The combination of ICIs with classical cytostatics, such as paclitaxel/carboplatin, and PLD or with PARPis did not significantly improve the results of treatment.

The first results of studies with immunotherapy of OC patients have shown that without a better understanding of the mechanisms of tumor immune escape, better characterization of the TME, and mechanisms related to immunotherapy resistance, continuing research in this cancer issue may end up similarly to studies with avelumab.

Table 3 provides an overview of the ongoing phase III clinical trials with ICIs.

## 6. Hypoxia Alleviation as the Condition to Combined Treatments against Ovarian Cancer 

In the TME, tumor cells represent the population that proliferates, moves, and escapes with the help of the TME conditions. This results from the cooperation with first, the blood vessels achieving the process of angiogenesis, together with the infiltrated immune/inflammatory cells [105] and various cells, such as cancer activated fibroblasts (CAFs) that compose, among others, “stromal cells” [106].

All cells associate in a cross-talk that dynamically participates in the evolution of the tumor in the TME context. This elaborated TME is consequently, determinant not only for the progression of a tumor but also for its aggressiveness. It exerts as a selection pressure to which tumor cells adapt through their heterogeneity [107] and similarly, heterogeneously react and resist when an anti-cancer treatment is applied. This is highly significant in the case of OCs. Indeed, besides the four main histological subtypes with low and high grades, OCs display a series of mutations and homologous recombination on germ lines, as well as epigenetic modifications. Such variability influences the stemness characters of cells, and consequently, the phenotype of ovary tumor initiating cells. In the case of OCs [108], in addition to the biological features of TME, the growing tumor mass generates hypoxia as a result of the physical lack of access to the oxygenated circulating blood. Hypoxia plays a maximal part as ovarian tumors develop and reside in a harsh hypoxic peritoneal environment, which additionally lowers the overall oxygen tension. Indeed, ascites aggravate hypoxia [109]. Consequently and more acutely in the development of OCs than other types of cancer, hypoxic stress is the most crucial factor, although still poorly taken into account in the design of treatment procedures.

It is remarkable that the main step determining the switch that turns a tumor from benign into an aggressive, uncontrolled growing tumor is the angiogenic switch (i.e., when the tumor goes from being physioxic to being hypoxic) [110]. This corresponds to signaling for angiogenesis, to bring in blood for reoxygenation, aiming to get back to the physioxic level of the normal ovarian tissue and transform the TME composition. In the tumor, angiogenesis never gets to be balanced because of the constant hypoxic state of the growing tumor mass [111]. Consequently, this maintains the need for neovascularization, which develops into a pathologic, non-efficient, and anarchic network. The resulting cellular and humoral components reflect an overall response, the origin of which is the hypoxic stress.

Thus, hypoxia of the TME is highly significant for the issue of cancer clinical treatment outcomes. More particularly, it determines the patients’ responses to treatments (especially to immunotherapy) because of its direct effect on metabolic changes, cell plasticity, and immunosuppression [112]. Indeed, cancer-linked immune resistance is a direct consequence of hypoxia-dependent shaping by the selection pressures exerted on the TME cells [113]. 

In that line, numerous immunoregulatory pathways render T cell-mediated tumor destruction inefficient. For example, the activity of killer immune cells in the immunological recognition of tumors is compromised by their concerted action with components of the tumor stroma, leading to immunosuppression [107].

This immunosuppressive conditioning of the TME affects the immune checkpoints orchestra. By their level of expression, distribution, and activity, the immune checkpoints molecules do cooperate to inactivate the tumor killer cells and tolerize tumor antigen-specific CD8 T cells [114]. Tregs are increased, and the phenotype of M1 macrophages is switched to M2 [115], actively increasing the overall immunosuppressive character of the tumor site.

As described above, very active blockers of immune checkpoints molecules are mainly antibodies directed to T cells and NK cells, such as anti-PD-1 [116] and anti-CTLA-4 [94,116], while anti-PD-L1 antibodies are mainly directed towards the tumor cells, endothelial cells, and macrophages in the TME. The modulation of their activity appears to be hypoxia-dependent. This may explain why using such blockers that improve survival in several types of metastatic cancers failed in a large proportion of patients with advanced cancer.

Those works are also pointing to the numerous and dynamic properties of the TME regulating the tumor cell response to immunotherapy. The unique-type of response is limited to the tumor-specific T cells. Thus, the tumor appears as a complex site composed as a whole “pseudo-organ” into which hypoxia regulates intercellular cooperation/recognition and dysregulates their effects comparatively to physioxia.

As a direct consequence, the hypoxic stress causes errors during the DNA repair process leading to mutations, and generally, to genomic instability that participates in the observed heterogeneity of the phenotypes displayed by cancer cells and their dynamic adaptation, described as plasticity. Because of such a permanent ability to adapt, plasticity is one of the features favoring the escape of tumor cells from susceptibility to treatment. This is particularly illustrated by the tumor cell’s property to undergo EMT. Distinct clones are produced during EMT with various degrees of ability to give rise to resistant and aggressive populations [117]. As a consequence, an effective treatment should not only take into account the tumor heterogeneity/plasticity to eliminate resistant cell selection but should also be devoted to avoiding sustained hypoxia.

Alleviating hypoxia is a challenging step and a crucial condition for treatment efficacy [118]. It is the criterion for adjuvant strategies designed to favor drug effects. Tumor neovascularization does not restore proper oxygenation despite the enhanced angiogenic activity. This makes vessel normalization approaches promising to enhance the potential of chemotherapy, as well as radiotherapy, and to overcome the main pitfalls met during immunotherapeutic treatments [111].

Normalized vessels allow for blood flow that mechanically helps treatments to reach the newly irrigated tumor cells. In addition to this physical role, the blood-borne oxyhaemoglobin dissociation in erythrocytes permits compensation of hypoxia, thus, breaking the chronic hypoxic stress [82,119,120].

The changes are effective on the whole microenvironment and indicate that combined treatments leading to elaborate adjuvant strategies are promising for immunotherapies [40,121,122]. Not only, but particularly illustrated, in the case of OCs, PD-1/PD-L1 recognition is very efficiently inactivating the immune response. This reaction is favored by hypoxia [123]. Indeed, PD-L1 expression has been shown to be induced by hypoxia in many cells of the TME and the tumor cells themselves. It was found in a circulating form and on key immune cells, such as dendritic cells (DCs) and macrophages [123,124]. 

In most cancers and particularly OC, the appearance of high-levels of PD-L1 corresponds to the angiogenic switch due to the establishment of hypoxia and occurs when the immunosuppression makes the treatments inefficient. In cancer and TME cells, the PD-L1 response to hypoxia is a final effect of the HIF-1-dependent induction of the PI3K/AKT/mTOR pathway [125]. Its activity is controlled upstream by the activity of the tumor suppressor PTEN, which is particularly important in OC [125].

Control of PD-L1 expression is directly exerted by the level of the local oxygen tension in the TME. In endothelial cells, PD-L1 induction by hypoxia is a hallmark of the pathologic angiogenesis on the endothelial cell membrane. Such overexpression correlates with the soluble form of PD-L1, which increases the PD-1 expression on the immunocompetent cells, such as NK cells and CD8T cells, making them both more susceptible to programmed death and blocking their passage into the tumor mass at the endothelial cell barrier level. Consequently, such a concordance of effects makes it necessary to design combinatorial treatments for OC. Strategies aim to modify the hypoxic situation by rendering the vessels functional to ensure the necessary blood flow to give the anti-PD-L1 antibodies access to tumor cells and other PD-L1+ cells of the TME. The latter compromise the action of PD-1+ immunocompetent cells, as NK and CD8T cells. As such, most of the combined treatment strategies are devoted to normalization of the vessels by using so-called anti-angiogenic monoclonal antibodies or molecules mostly directed to neutralize the excess of VEGF produced by the hypoxic tumor cells [121,122,126].

The challenge is the proper balance to avoid the deep anti-angiogenic effect leading to vessel destruction, anoxia, and selection of resistant cancer stem cells [127]. Indeed, in ovary cancer patients, PD-L1 expression indicates a bad prognosis and low treatment outcomes [128].

**Figure 2:**
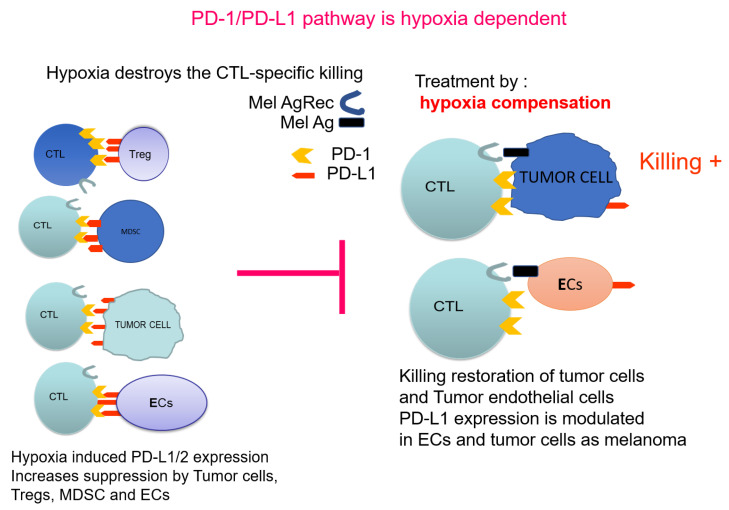
PD-1/PD-L1 pathway as hypoxia dependent: Effect on immunosuppression. 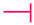 indicates the blocking effect of hypoxia-induced PD-L1 towards normal killing.

In conclusion, future approaches will aim to combine methods, which will first modify the TME by alleviating hypoxia to allow the further application of downstream combinations of anti-cancer therapies. Figure 2 illustrates PD-1/PD-L1 pathway in the context of hypoxia. The properties of the cancer endothelial cells and their response to hypoxia, indicate that PTEN-mediated regulation of PD-L1 is a most promising adjuvant approach. PTEN, as a controller of the angiogenic process, is a target to increase the chances of further immunotherapies.

The mechanisms of resistance display a large spectrum of complexity in limiting the efficacy of checkpoint inhibitor monotherapy, combinatorial approaches are crucial. Monitoring angiogenesis is the most direct and non-avoidable condition to modify the properties of TME, so that tumor tolerance and resistance, can be overcome.

Again, hypoxia alleviation appears necessary for its influence on T cell function. Consequently, the combinatorial strategies focused on angiogenesis normalization are necessary to allow for efficient immunotherapy through its effect on both ICIs and induction of immune cells response. Such conditions, when fulfilled, will permit us to efficiently apply other therapies without facing failures that are actually encountered and that are attributed to the TME protection of the tumor expansion.

TME should be considered as an important target for anti-cancer therapy, allowing patients to ultimately benefit from drug combinations.

## 7. Conclusions

Hypoxia plays a crucial role in modulating the response of cells to various drug treatments. It is one of the key factors contributing to the observed chemoresistance. Targeting HIF-1 emerges as a tool for increasing the effectiveness of standard chemotherapy and helping to overcome chemoresistance. Thus, treatments directed at HIF, and consequently, controlling VEGFs, should help regulate angiogenesis and change the microenvironment.

It is to be noticed that, although treatments were devoted to tumor cells, their efficacy cannot be dissociated from their effect on the other cells of the microenvironment. Namely, cells that enter the tumor to kill it.

Immunotherapeutic strategies, having undoubtedly opened a new era in cancer research and treatment, are the best example of a need for the knowledge of the local conditions in which they have to operate. The typical example is provided by the anti-angiogenic therapies. Most of them aim at regulating the excessive growth of vessels, such as monoclonal antibodies against VEGFs or other stimulators of endothelial VEGF receptors [129]. Most often, they are designed to neutralize the excess VEGF produced in response to tumor hypoxia. Their highly successful efficacy leads to the destruction of newly formed vessels. Instead of making tumor cells apoptotic because of the lack of O_2_ and blood-borne nutrients, the whole process makes the cells resistant. Dedifferentiation is amplified, and cancer cells are selected to form a stem-like cell population (quiescent and senescent) but, at the same time, susceptible to move into an aggressive and highly invasive cell population. This raised the need for vessel normalization strategies, which appear to be the main present challenge that anti-cancer research has to face. Based on the cited knowledge of the local tumor conditions (i.e., the microenvironment), the normalization of the pathologic vessels is the main approach with the potential to reach a complete modification of the tumor environmental properties both in its molecular and cellular components [93,130]. The failure of recent clinical trials of ICIs naturally raises questions about the reason for such a phenomenon. One possible explanation emerges considering the hypoxia effect on TME. It was shown that HIF-1α directly increased PD-L1 gene expression in MDSCs, macrophages, dendritic cells, and tumor cells [124]. Lymphatic endothelial cells caused systemic peripheral tolerance through the expression of PD-L1. Observed tolerance occurred because of the lack of co-stimulation resulting in high-level PD-1 expression on CD8 T cells [131]. Therefore, concurrent PD-L1 and high-level PD-1 expression may provide a novel approach for immunotherapy in OC. Moreover, a better understanding of the mechanism involved in OC. Moreover, a better understanding of the mechanisms involved in OC hypoxia may provide biomarkers that would allow reliable monitoring and assessment of the efficacy of immunotherapy.

For efficient therapies against tumors, it appears that the reversal of hypoxic features brings promise to invert the immune suppression into active immune response. This type of approach should be understood, as an adjuvant mean to permit the application of treatments and provide a high added therapeutic value.

## Figures and Tables

**Table 1:** Molecular targets of drugs applied in ovarian cancer treatment.

Drug	Type of Drug	Targets/Mode of Action	Type of Cells
Cisplatin/Carboplatin	Platinum-based chemotherapeutic Compound	DNA (alkylating DNA/cross-linking DNA, induction of mispairing of nucleotides)	Cancer cells
Paclitaxel (taxanes)	Mitotic inhibitor	Beta subunit of tubulin (prevents its polymerization into microtubules)	Cancer cells
Cyclophosphamide	Alkylating agent	Guanine residues	Cancer cells
Topotecan		Topoisomerase I (it inhibits its action)	Cancer cells
Doxorubicin	Antibiotic	DNA, Topoisomerase II	Cancer cells
Bevacizumab	Monoclonal antibody	VEGF-A	Cancer cells
Olaparib	PARP inhibitors	PARP	Cancer cells
Avelumab	Immune checkpoint inhibitor (antibody)	PD-L1	Cancer cells/endothelial cells, myeloid-derived suppressor cells, M1 macrophages
Pembrolizumab	Immune checkpoint inhibitor (antibody	PD-1	T lymphocytes, Tregs, NK cells, Th2

**Table 2:** Summary of research on ovarian cancer (OC) cells reaction to chemotherapy treatment in hypoxia or related to the hypoxia pathway.

Agent	Cells/Tissue	Type of Hypoxia	Main Findings	Ref
**Platinum-Based Compounds**				
Carboplatin	ES2, OVCAR3, OVCAR8, A2780 and A2780cisR	CoCl_2_	Cysteine was beneficial for A2780isR cells upon carboplatin exposure ES2 and OVCAR8 cells depended more on cysteine when treated with carboplatin in hypoxia than OVCAR3 cells In patients, of total and S-homocysteinylated levels distinguished blood donors from patients and patients with benign tumors from those with malignant disease	[72]
Carboplatin	ES2 and OVCAR3	CoCl_2_	Cells in hypoxia proliferated less, but their survival rate was higher in adverse conditions: Cells in normoxia proliferated rapidly, but their survival rate was decreased in adverse conditions Cysteine affected the adaptation of cancer cells to CoCl_2_-mimicked hypoxia and hypoxia-derived platinum-based chemotherapy resistance, resulting in the selection of more aggressive phenotypes	[71]
Cisplatin	A2780, A2780/CP, PEO1, and PEO4	No hypoxia	Cisplatin downregulated the level of hypoxia-inducible factor-1α (HIF-1α) by increasing its degradation) in cisplatin-sensitive OC cells but not in their cisplatin-resistant counterparts Overexpression of a degradation-resistant HIF-1α reduced cisplatin-induced apoptosis in cisplatin-sensitive cells, Disabling HIF-1α augmented the response to cisplatin in both cisplatin-sensitive and cisplatin-resistant OC cells (mechanism of the latter one is the redirection of aerobic glycolysis in the resistant cancer cells towards mitochondrial oxidative phosphorylation and reactive oxygen species (ROS) overproduction)	[50]
Cisplatin	Cisplatin-sensitive (A2780) and cisplatin-resistant (A2780cis)	Hypoxia chamber 0.5%	Treatment with cisplatin in hypoxia was the most important factor determining chemoresistance, and exposing both cell lines to chronic or acute hypoxia prior to the treatment enhanced the resistance ANGPTL4 and HER3-novel markers of hypoxia	[52]
Cisplatin	IOSE, OVCAR, A2780, TR127 and TR182	Hypoxia chamber 1%	Co-culture of hypoxic exosomes (Hex) with tumor cells decreased DNA damage and increased cell survival in response to cisplatin treatment Exosomes from patient-derived OC cell lines (of ascitic origin) cultured under hypoxic conditions carried oncogenic proteins, namely, STAT3 and FAS (capable of increasing cell migration/invasion, chemoresistance, and tumor progression/metastasis)	[54]
Cisplatin	SKOV3, OVCAR3, PA1 and IOSE385	<1% Anaerobic system model 1025 (chamber)	Hypoxia-derived ROS induces mitochondrial fission and cisplatin resistance via downregulation of proteins involved in fission and fusion (p-Drp1 and Mfn1) in OC cells, and their inhibition enhanced the chemosensitivity of OC cells in hypoxia hypoxia induces cisplatin resistance in OC cells irrespective of their p53 status	[53]
Cisplatin	SKOV3	Hypoxia chamber 1%	Hypoxia enhanced both the expression of formyl peptide receptor (FPR) and toll-like receptor 9 (TLR9), as wells as stimulate the release of ligands for both receptors in human OC cell line SKOV3	[70]
Cisplatin	OVCAR3	Hypoxia chamber 1%	Hypoxia inhibited cisplatin-induced apoptosis in OVCAR-3 cells and enhanced their chemoresistance to cisplatin, which was associated with HIF-1α-induced autophagy	[55]
Cisplatin	SKOV3	CoCl2 (200 µM)	*Scutellaria baicalensis* (SB) treatment decreased HIF-1α expression in cancer cell lines, and the effect of this treatment was similar to the cycloheximide inhibitors PI3K-LY294002 and MAPK-PD98059 SB treatment reduced activation of PI3K/AKT and MAPK/ERK seen in OC cells SB increased the effects of cisplatin on OC cells by reducing the expression of HIF-1α, ABCG1, and ABCG2	[59]
Cisplatin	ovarian cancer tissues, C13* and OV2008 cell lines	-	Compared with the corresponding normal tissues, in the ovarian tumors, miR-199a expression was decreased, and HIF-1α was increased Compared with C13* cells, on OV2008 cells, the expression of miR-199a was higher In OV2008 cells, attenuation of HIF-1α reversed the inhibiting function of the miR-199a inhibitor on cisplatin-induced apoptosis. In C13* cells, the overexpression of both miR-199a and HIF-1α reduced cisplatin-induced apoptosis, and miR-199a may change cisplatin resistance in OC cells by regulating HIF-1α	[66]
Cisplatin	A2780 and SKOV3	No chamber	Rab25 regulates HIF-1α protein expression in an oxygen-independent manner in OC cell lines by *de novo* protein synthesis (via the Erbb2/ERK1/2 and p70S6K/mTOR pathways), not by enhancing transcription Rab25 expression enhanced cisplatin resistance and conferred intraperitoneal growth to the A2780 cell line (immunocompromised mice) Targeting HIF1 activity (HIF-1β) re-sensitized cells to cisplatin in vitro and reduced tumor growth (of A2780-Rab25 expressing cells) in vivo; similar results were achieved for SKOV3 cells (which express endogenous Rab25 and HIF-1α in normoxia)	[69]
Cisplatin	SKOV3	CoCl_2_	SENP1 enhanced the expression of HIF-1α by deSUMOylation and decreased the sensitivity of hypoxic SKOV3 cells to cisplatin	[67]
Cisplatin	RMG2/JHOC5 and RMG2/JHOC5 knockdown cells	Hypoxia chamber 2%	Hepatocyte nuclear factor 1 homeobox B (HNF1β), which is expressed in clear cell OC (not other subtypes), enhances aerobic glycolysis, and increases glutathione (GSH); a process that is probably regulated by rBAT, one of the cystine transporters	[84]
Cisplatin Natural compound	A2780, adriamycin-resistant A2780/ADR and cisplatin- resistant A2780/CP cell lines.	Hypoxia chamber 2%	Sulforaphane In hypoxia, modulates several oncogenic factors via enhancing anti-cancer reaction (p53, ARE, IRF-1, Pax-6, and XRE) and suppressive actions maintaining tumor progression (AP-1 and HIF-1). lowers the level of HIF-1α protein (not affecting its transcription/stability) can decrease the level of CA IX translation and transcription (HIF-1 target, which preserves cancer cells from hypoxia-related pH imbalance and promotes their migration/invasion) leads to decreased pH control and lowered migration of OC cells	[65]
Cisplatin	SK-OV-3, OVCAR-3, and HEY cells, pancreatic cells (MIA PaCa-2, BxPC-3 and ASPC-1)	Hypoxia chamber 1%	Pretreatment with SB202190 (p38 MAPK inhibitor) increased the activity of both in selected cancer cell lines Pretreatment with SB202190 increased the actions of both 2-deoxy-glucose (2-DG) and D-allose with platinum analogs in the majority of tested cell lines HIF-1α protein activity and accumulation was lowered by SB202190 SB202190 induced sensitization of tumor cells to 2-DG and D-allose, which may be partially mediated by inhibition of HIF-1α activity; combining glucose analogs and p38 MAPK inhibitors with chemotherapy may be an effective approach to target glycolytic tumor phenotypes	[60]
Cisplatin	OVCAR3, SW626, and normal human placental cells (HS 799 pl)	No hypoxia	Amla Extract (AE) and quercetin (AE component) significantly enhanced the in vitro expression of the autophagic proteins (beclin1 and LC3B-II); AE didn’t cause apoptosis AE decreased expression of HIF-1α and other angiogenic genes in OVCAR3 cells; similar effects observed in vivo (in tumor xenografts) were accompanied by reduced endothelial cell antigen expression AE with cisplatin decreased cell proliferation and enhanced autophagic protein expression *in vitro* Probable mechanism of AE action—induction of autophagy and inhibition of angiogenesis	[58]
Cisplatin	A2780	<2% (*in vivo*)	Phosphorylation of STAT3 (Tyr705) in A2780 cancer cells was increased upon exposure to hypoxia Such hypoxic cancer cells were more resistant to standard chemoterapeutics and targeting STAT3 increased number of apoptotic cells after anti-cancer treatment	[85]
Cisplatin	HEY, SKOv3, and MDA-MB-231	CoCl_2_	Chemically induced hypoxia helped to select polyploid giant cancer cells (PGCCs) from human OC cell lines and primary OC PGCCs: Expressed normal and cancer stem cell markers; divided asymmetrically and cycled slowly; and differentiated into adipose, cartilage, and bone Single PGCCs formed cancer spheroids in vitro and generated tumors (of a mesenchymal phenotype, more resistant to cisplatin) in immunodeficient mice	[86]
Cisplatin	A2780 and A2780CP	No hypoxia	Fasudil, an inhibitor of the Rho/ROCK pathway, inhibited HIF-1α expression and augmented growth inhibition and apoptosis caused by cisplatin in OC cells	[61]
Cisplatin	A2780 (wt TP53) and OVCAR3 (mutated TP53)	No hypoxia	Cisplatin-induced expression of angiogenesis-related genes, including HIF-1α but not VEGF	[49]
Cisplatin	C13K	CoCl_2_ (200 µM)	Noscapine made chemoresistant (due to cobalt induced hypoxia) OC cells sensitive to cisplatin-induced apoptosis and inhibition of cell proliferation Noscapine induced proteasome-mediated degradation of cobalt-stabilized HIF-1α protein, (decreasing its transcriptional activity)	[62]
Cisplatin	A2780 and OVCAR	Hypoxia chamber, no info on %	Cell lines showing resistance to cisplatin increased RON expression Hypoxia increased RON expression in OVCAR-3 cells, decreasing E-cadherin gene expression	[87]
Cisplatin	A2780, SKOV3, and OVCAR	Hypoxia chamber 1%	Cisplatin and doxorubicin repressed hypoxic induction of VEGF expression by inhibiting HIF-1 through different mechanisms. cisplatin strongly reduced the protein levels of the HIF-1 co-activators p300 and CREB-binding protein (CBP) under hypoxia	[51]
**Alkylating Agents**				
Cyclophosphamide (CPA, Cytoxan)	LS174T and T47D	No hypoxia chamber, but HRE	Macrophages transduced with an adenoviral vector expressing cytochrome CYP2B6 (under synthetic hypoxia-responsive elements) during CPA treatment exhibited increased cytotoxicity against several cancer cell lines compared to untransduced macrophages or macrophages transduced with CYP2B6 alone. In human OC xenograft mouse models, animals treated with transduced macrophages plus CPA lived up to 2 times longer than mice treated with untransduced macrophages and CPA	[73]
Melphalan	RPMI8226 (myeloma)	No hypoxia	When investigated in myeloma cells, the drug applied in OC treatment, increased their survival partially via VEGF- and IL8-induced PI3K/p38 signaling, not investigated in hypoxia	[74]
**Mitotic Inhibitors**				
Paclitaxel	A2780, SKOV3, and OVCAR	Hypoxia chamber 1%	Paclitaxel (and docetaxel) neither affected VEGF expression nor HIF-1 activity	[51]
Albumin bound paclitaxel (nab-paclitaxel)	SKOV3ip1, HeyA8, and HeyA8-MDR		Treatment with nab-paclitaxel alone and combined with topotecan (both applied in a metronomic manner) resulted in lower mice tumor weight compared with vehicle alone Combination metronomic therapy (vs. other therapies): (1) Decreased tumor weight in the HeyA8-MDR model; (2) increased overall survival of mice, and (3) resulted in the highest reduction of microvessel density and proliferation Treatment of endothelial cells with conditioned (with metronomic combination therapy or nab-paclitaxel alone) media decreased their ability to form tubes	[79]
Paclitaxel	A2780 and SKOV3	Hypoxia chamber 1%	Hypoxia decreased paclitaxel-induced G2/M phase arrest, probably due to G1 phase promotion c-Src supported hypoxia-related paclitaxel resistance in human OC cells by decreasing G2/M phase arrest, and it’s abolishment (with FV-429) reversed resistance by inhibiting the Src/STAT3/HIF-1α pathway	[77]
Paclitaxel	OVCAR-3, MDAH-2774 and SKOV-3	No hypoxia	sMEK1 and paclitaxel decreased phosphorylation of S6K and 4E-BP (downstream targets of the mTOR-signaling pathway) and expression of their (S6K and 4E-BP) downstream targets, e.g., HIF-1α and VEGF (in vitro and in vivo)	[78]
Paclitaxel	Epo-treated A2780 and SKOV-3 cells	Hypoxia chamber 1%	Hypoxically conditioned media of Epo-treated A2780 stimulated human umbilical vein endothelial cells (HUVECs) Such conditioned media (hypoxia and Epo-treated A2780) increased phosphorylation of STAT-5 in HUVECs	[76]
Paclitaxel	A2780	Hypoxia chamber 1%, 3%, and 5%	HIF-1α increased in hypoxia, contributing to chemoresistance by G0/G1 arrest	[75]
**Antibiotics**				
Doxorubicin	A2780, SKOV3, and OVCAR	Hypoxia chamber 1%	Doxorubicin inhibited hypoxic induction of VEGF expression by inhibiting the activity of HIF-1, but it didn’t affect the expression of HIF-1α and only weakly decreased hypoxic expression of p300 and CBP	[51]
**Miscellaneous Antineoplastics**				
Topotecan	CAOV3 CAOV4 and OVCA429	Hypoxia chamber 0.5% and 1%	Drug resistance in hypoxia was associated with HIF-1α binding to and decreasing p53 transcriptional activity of OC cells Topotecan mechanism of action involved TOPO1 locating onHIF1α mRNA and resulted in restoring the activity of p53, diminishing expression of ABCB1/ABCB5, and annulling resistance of cisplatin and paclitaxel in hypoxia	[83]
Topotecan	SKOV3, Hey8a, and Hey8a-MDR; female athymic mice (NCr-nu)		Topotecan inhibited HIF-1α and increased hypoxic areas in mice	[79]
Metronomic topotecan	HeyA8 and SKOV3ip1	CoCl_2_ (250 μM)	Topotecan decreased levels of HIF-1α and VEGF in OC cells (HeyA8 and SKOV3ip1) independently from proteasome degradation and topoisomerase I	[81]

**Table 3:** Immune checkpoint inhibitors in ongoing phase III clinical trials for ovarian cancer.

Drug	Molecular Target	Line of Treatment	Schema of Randomization	Control Arm	Study Arm	Primary End Points	ClinicalTrials.gov Identifier
Atezolizumab	PD-L1	1st, 2nd, 3rd relapse after platinum-based therapy < 6 months	1:1	Chemotherapy: Paclitaxel 80 mg/m^2^ d1, 8, 14, 22 q28 or pegylated liposomal doxorubicin (PLD) 40 mg/m^2^ q28 + Bevacizumab 10 mg/kg q14 + Placebos q14	Chemotherapy: Paclitaxel 80 mg/m^2^ d1, 8, 14, 22 q28 or PLD 40 mg/m^2^ q28 + Bevacizumab 10 mg/kg q14 + Atezolizumab 840 mg q14	PFS, OS	NCT03353831
Atezolizumab	PD-L1	1st, 2nd relapse after platinum-based therapy >6 months	1:2	Placebo 1200 mg × 6 cycles q3 wk or 800 mg × 6 cycles q4 wk during treatment with platinum-based chemotherapy and bewacizumab, followed by placebo 1200 mg q3 wk until progression	Atezolizumab 1200 mg × 6 cycles q3 wk or 800 mg × 6 cycles q4 wk during treatment with platinum-based chemotherapy and bewacizumab, followed by atezolizumab 1200 mg q3 wk until progression	PFS	NCT02891824
Atezolizumab	PD-L1	1st, 2nd, relapse after platinum-based therapy >6 months	1:1	Placebo of atezolizumab in combination with platinum-based regimens followed by maintenance niraparib with placebo	Atezolizumab in combination with platinum-based regimens, followed by maintenance niraparib with atezolizumab	PFS	NCT03598270
Atezolizumab	PD-L1	1st-line FIGO III/IV	1:1	Paclitaxel, carboplatin, placebo of atezolizumab and bevacizumab, followed by maintenance therapy bevacizumab with placebo of atezolizumab for a total of 22 cycles of atezolizumab and 21 cycles of bevacizumab	Paclitaxel, carboplatin, atezolizumab and bevacizumab, followed by maintenance therapy bevacizumab with atezolizumab for a total of 22 cycles of atezolizumab and 21 cycles of bevacizumab	PFS, OS, OS based PD-L1 status	NCT03038100
Atezolizumab	PD-L1	1st, 2nd, relapse after platinum-based therapy <6 months	1:1:1	Arm 3: PLD on day 1 and bevacizumab on days 1 and 15	Arm 1: PLD on day 1 and atezolizumab on days 1 and 15 Arm 2: PLD on day 1, bevacizumab on days 1 and 15, and atezolizumab intravenous (IV) on days 1 and 15	OS	NCT02839707
Pembrolizumab	PD-1	1st-line FIGO III/IV	1:1:1	Arm 3: Carboplatin/paclitaxel PLUS placebo for pembrolizumab on Day 1 of each 3-week cycle for up to 35 cycles PLUS placebo for olaparib via oral tablet twice each day (BID), starting with Cycle 7.	Arm 1: Carboplatin/paclitaxel PLUS pembrolizumab 200 mg on Day 1 of each 3-week cycle for up to 35 cycles PLUS olaparib 300 mg via oral tablet (BID), starting with Cycle 7. Arm 2: Carboplatin/paclitaxel 3-week cycles starting in Cycle 1 PLUS pembrolizumab 200 mg via IV infusion on Day 1 of each 3-week cycle for up to 35 cycles PLUS placebo for olaparib via oral tablet BID, starting with Cycle 7.	PFS, OS	NCT03740165
Nivolumab	PD-1	Maintenance treatment following response to 1st-line platinum-based chemo-therapy	1:1:1:1	Oral placebo + IV placebo	Arm A: Oral rucaparib + IV nivolumab Arm B: Oral rucaparib +I V placebo Arm C: Oral placebo + IV nivolumab	PFS	NCT03522246
Durvalumab	PD-L1	1st-line FIGO III/IV	1:1:1:1	Arm A: Platinum-based chemotherapy in combination with bevacizumab and durvalumab placebo, followed by maintenance bevacizumab, durvalumab placebo, and olaparib placebo	Arm B: Platinum-based chemotherapy in combination with bevacizumab and durvalumab, followed by maintenance bevacizumab, durvalumab, and olaparib placebo. Arm C: Platinum-based chemotherapy in combination with bevacizumab and durvalumab, followed by maintenance bevacizumab, durvalumab, and olaparib. Arm tBRCAm cohort: Platinum-based chemotherapy in combination with bevacizumab and durvalumab, followed by maintenance bevacizumab, durvalumab, and olaparib	PFS	NCT03737643

The above studies have not yet presented conclusive results, although data on projects and planned endpoints are available.

## Abbreviations

PDS	primary deburring surgery
NACT	neoadjuvant chemotherapy
OS	overall survival
PFS	progression-free survival
FIGO	The International Federation of Gynecology and Obstetrics)
HIPEC	hyperthermic intraperitoneal chemotherapy
PARP	Poly(ADP-ribose)polymerases
BRCA1	Breast cancer type 1 susceptibility protein
EMT	epithelial-to-mesenchymal transition
HIF-1α	hypoxia-inducible factor-1 alpha
pVHL	Von Hippel Lindau protein
VEGF	vascular endothelial growth factors
MMP2	metalloproteinase 2
UPAR	angiopoietin 2 and urokinase receptor
DSF	disease-free survival
CSS	cancer-specific survival
RFS	relapse-free survival
MFS	metastasis-free survival
CBP	CREB-binding protein
HEx	hypoxic exosomes
NEx	normoxic exosomes
PD-1	programmed death protein
PD-L1	programmed death ligand 1
XRE	X-responsive elements
rhFSH	human recombinant follicule stimulating hormone
ROS	reactive oxygen species
IL8	Interleukin 8
Epo	erythropoietin
RTK	receptor tyrosine kinase
HRE	hypoxia-responsive elements
AE	amla extract
HRR	homologous recombination repair
FAK	focal adhesion kinase
PDGFR	platelet-derived growth factor receptor
PP2A	protein phosphatase 2A
CTLA-4	cytotoxic T lymphocyte-associated protein 4
DCR	disease control rate
CAF	cancer activated fibroblasts
TME	tumor microenvironment
PTEN	phosphatase and tensin homolog deleted on chromosome ten
NK	natural killers
MDSCs	myeloid-derived suppressor cells
ECs	endothelial cells
